# Novel Multi-Ingredient Supplement Facilitates Weight Loss and Improves Body Composition in Overweight and Obese Individuals: A Randomized, Double-Blind, Placebo-Controlled Clinical Trial

**DOI:** 10.3390/nu15173693

**Published:** 2023-08-23

**Authors:** Joshua P. Nederveen, Alexander J. Mastrolonardo, Donald Xhuti, Alessia Di Carlo, Katherine Manta, Matthew R. Fuda, Mark A. Tarnopolsky

**Affiliations:** 1 Department of Pediatrics, Faculty of Health Sciences, McMaster University Medical Center (MUMC), Hamilton, ON L8N 3Z5, Canada; nedervj@mcmaster.ca (J.P.N.);; 2 Exerkine Corporation, McMaster University Medical Center (MUMC), Hamilton, ON L8N 3Z5, Canada

**Keywords:** nutraceutical, catechin, polyphenol, chlorogenic acids, biomarker, multi-ingredient supplement

## Abstract

Background: Despite the growing recognition of the obesity crisis, its rates continue to rise. The current first-line therapies, such as dietary changes, energy restriction, and physical activity, are typically met with poor adherence. Novel nutritional interventions can address the root causes of obesity, including mitochondrial dysfunction, and facilitate weight loss. Objective: The objective of this study was to investigate the effects of a multi-ingredient nutritional supplement designed to facilitate mitochondrial function and metabolic health outcomes over a 12 wk period. Methods: Fifty-five overweight and/or obese participants (age (mean ± SEM): 26 ± 1; body mass index (BMI) (kg/m^2^): 30.5 ± 0.6) completed this double-blind, placebo-controlled clinical trial. Participants were randomized to 12 wks of daily consumption of multi-ingredient supplement (MIS; *n* = 28; containing 50 mg forskolin, 500 mg green coffee bean extract, 500 mg green tea extract, 500 mg beet root extract, 400 mg α-lipoic acid, 200 IU vitamin E, and 200 mg CoQ10) or control placebo (PLA, *n* = 27; containing microcrystalline cellulose) matched in appearance. The co-primary outcomes were bodyweight and fat mass (kg) changes. The secondary outcomes included other body composition measures, plasma markers of obesity, fatty liver disease biomarkers, resting energy metabolism, blood pressure, physical performance, and quality of life. The post-intervention differences between MIS and PLA were examined via ANCOVA which was adjusted for the respective pre-intervention variables. Results: After adjustment for pre-intervention data, there was a significant difference in weight (*p* < 0.001) and fat mass (*p* < 0.001) post-intervention between the PLA and MIS treatment arms. Post-intervention weight and fat mass were significantly lower in MIS. Significant post-intervention differences corrected for baseline were found in markers of clinical biochemistry (AST, *p* = 0.017; ALT, *p* = 0.008), molecular metabolism (GDF15, *p* = 0.028), and extracellular vesicle-associated miRNA species miR-122 and miR-34a in MIS (*p* < 0.05). Conclusions: Following the 12 wks of MIS supplementation, weight and body composition significantly improved, concomitant with improvements in molecular markers of liver health and metabolism.

## 1. Introduction

The obesity epidemic continues to grow unabated on a global scale. Over the last 30 years, the incidence of obesity has nearly tripled [1,2], with body mass index (BMI) continuing to trend upward [3,4]. In North America, ~70% of the adult population is overweight, with ~30% classified as obese (defined as BMI ≥ 30 kg/m^2^) [5,6,7]. Emergent countries appear to be growing in risk as well, with evidence suggesting that an obesogenic environment exists in both poor and affluent populations alike [8]. Obesity exacerbates the pathology of many other conditions, including type 2 diabetes, non-alcoholic fatty liver disease (NAFLD), a variety of cancers, cardiovascular disease, poor mobility, and all-cause mortality [9,10,11,12,13,14].

Ultimately, the development of obesity can stem from an increased energy intake concomitant with reduced energy expenditure [15]. An overwhelming percentage of the North American population does not meet the minimum guidelines for physical activity [16], and evidence suggests that individuals find exercise to be inconvenient, costly, challenging, or otherwise unenjoyable [17]. Consuming a healthy, well-balanced diet without excessive proportion size is a method of reducing energy intake to promote fat loss and maintain a state of good health [18]; however, there are many socioeconomic barriers to eating a healthy diet, such as a lack of access to, greater cost of, and greater time commitment required to prepare it, as well as the unpalatability of many healthy foods or meals in comparison to less healthy alternatives [19].

Another factor in the etiology of obesity is excessive energy intake, which can cause oxidative stress [20,21] and, subsequently, mitochondrial dysfunction. While mitochondria are a crucial aspect of metabolism and the production of ATP from food substrates, they are also a major source of reactive oxygen species (ROS) in the cell [22,23]. Obesity-induced alterations in mitochondrial function can subsequently lead to increases in ROS production [20,24,25]. The increased oxidative stress generated by mitochondrial dysregulation can in turn promote aberrant cytokine production [26], which can contribute to the phenotype of chronic, low-grade inflammatory profile prevalent in obesity [27,28]. Further, some of these cytokines, peptides, and other factors such as microRNAs [29,30,31], known to play a role in the development of obesity, have been shown to be enclosed within extracellular vesicles [32]. Due to this important bioactive content, extracellular vesicles (EVs) have been implicated in the development and propagation of obesity [33,34]. Altogether, obesity is a multifactorial disease, with roots in mitochondrial function, upregulated ROS production, and the dysregulation of circulating inflammatory factors and bioactive EVs.

To address the constellation of factors underlying obesity, a multi-faceted approach must be taken. To counteract the cycle of ROS-associated weight gain, mitochondrial dysfunction, and subsequent inflammation, several natural ingredients have exhibited antioxidant properties. Supplementation with coenzyme Q10, an intermediate in the electron transport chain (ETC), has been shown to rescue pro-oxidative markers in serum and lower lipid peroxidation [35,36]. Similarly, fat-soluble vitamin E [37,38,39] and α-lipoic acid (α-LA) [40,41] have been shown to facilitate improved antioxidant status. Nitrate-rich beetroot extract (derived from the root of *Beta vulgaris*) can improve the efficiency of mitochondria [42] and has been shown to have considerable antioxidant effects [43,44,45]. These ingredients, in combination with other nutraceuticals known to stimulate weight loss via a variety of biological pathways, such as forskolin (derived from *Coleus forskohlii*) [46,47], green coffee bean [48,49], and green tea (derived from the leaves of *Camellia sinensis*) extract [50], have been associated with improving body composition [51] and may simultaneously influence the main pathways underlying obesity.

While these ingredients have often been examined individually, there is limited utilization regarding the potential for a multi-ingredient supplementation strategy targeting the multiple pathways known to be involved in the pathogenesis of obesity. Our pre-clinical data suggested that such a strategy lowered body fat, preserved muscle mass, and improved mitochondrial function in white adipose tissue [52]. Therefore, the primary aim of this study was to examine the effects of a multi-ingredient, metabolism-enhancing supplement (comprising seven ingredients, termed ‘MIS’) on weight management in overweight and/or obese individuals compared to the placebo control. We also examined exploratory outcomes: circulating biomarkers such as EV-enclosed miRNA as well as markers of fatty liver disease, glucose regulation, and inflammation. We hypothesized that the multi-ingredient nutritional intervention would decrease fat mass, preserve muscle mass and function, and reduce clinically relevant circulating biomarkers.

## 2. Materials and Methods

### 2.1. Participant Screening & Recruitment

Overweight and obese men and women were recruited for the current study. Potential participants were first screened by email or telephone to confirm that they were a male or female between the ages 18 and 50 y, were overweight (BMI 25–29.9 kg/m^2^) or obese (BMI > 30 kg/m^2^), and had no serious exercise contraindications for physical testing. Exclusion criteria included smoking, use of assisted walking devices, chronic use of analgesic or anti-inflammatory drugs, diagnosed diabetes mellitus, cardiovascular disease (recent myocardial infarction and/or medications hypertension requiring more than 2 medications), congestive heart failure, renal disease, previous stroke, active musculoskeletal injury and/or severe osteoarthritis, significant weight change in the 12 wk period prior to the study (no weight gain or loss >5 kg in the prior 12 wks), muscular dystrophy, severe peripheral neuropathy, severe osteoporosis, initiating medications known to affect protein or energy metabolism, a chronic obstructive pulmonary disorder, uncontrolled asthma and/or asthma requiring more than 2 medications, and participation in another biomedical study and/or structured exercise training regime within 4 wks prior to the initiation of intervention. A flowchart of the enrollment process is shown in Supplemental Figure S1. Those who qualified upon initial screening were invited to the Neurometabolic Clinic in the Department of Pediatrics (Faculty of Health Science) located in McMaster Children’s Hospital to confirm eligibility by completing the following: (i) a medical screening questionnaire; (ii) a complete physical activity readiness questionnaire; (iii) anthropometric measurements (e.g., height, weight, waist circumference), which was then used to calculate body mass index (BMI) for the purpose of classifying individuals as overweight or obese [53] and to calculate the waist-to-height ratio (WHR), as an indicator or measure of health status [54,55]. Participants completed the SF-36 Health Questionnaire [56]. Sixty-five overweight/obese men and women participated in this randomized, double-blind, placebo-controlled parallel group trial, which took place between March 2019 and April 2020.

### 2.2. Experimental Design

Participants were randomly allocated to receive either the multi-ingredient nutritional supplement (MIS) consisting of 7 ingredients (Table 1) or a control placebo (PLA) for the 12 wk intervention period using minimization [57,58]. Participants, as well as investigators who were responsible for recruiting and/or testing participants, were blinded to the individual group assignments. The MIS and PLA were identical in appearance, produced in a Good Manufacturing Practice (GMP), and coded and distributed by an independent third party licensed by Health Canada (Infinite Nutrition, Windsor, ON, Canada). The group randomization code was provided to the investigators by Infinite Nutrition following the trial. Study products were packaged and labeled in a blinded fashion by Infinit Nutrition Canada (Windsor, ON, Canada), in which both products were encapsulated in an opaque casing. To maintain the double-blind nature of the experiment, the placebo (PLA), which contained microcrystalline cellulose, was matched in size and appearance. Anthropometry data lock and the final locked data with randomization codes were simultaneously provided to an external third party. Infinit Nutrition did not have any access to the data.

The following assessments were performed on eligible participants at the initiation of the study and after completion of the 12 wk supplement period: DXA scan, blood pressure, antecubital blood draw, grip strength, aerobic capacity (VO_2max_), resting metabolic rate (RMR), 3-day dietary recall, and 1 wk (i.e., 7-d) pedometer step count. Dietary intake tracking instructions (to perform a 3D dietary recall or ‘food record’) were provided to the participants. Participants were given a standard pedometer (Omron HJ-321, Omron, Kyoto, Japan) and recorded their daily totals for 1 wk as an indicator of baseline activity level, concomitant with a self-reported ‘minutes of physical activity per day’ evaluation.

To assess the progress between study initiation and completion (i.e., 12 wk), participants returned for testing at the 2 wk and 5 wk mark from the study initiation to perform a DXA scan and anthropometric measurements. Weekly phone calls and/or emails were conducted throughout the study period to ensure participant compliance. At the onset of the study, all participants received a copy of the Canada Food Guide and the evidence-based Canadian Society for Exercise Physiology (CSEP) Movement Guidelines [59]. During the entire intervention period, participants were neither encouraged nor discouraged to maintain their initial body weight and physical activity level. The general experimental design is presented schematically in Supplemental Figure S2.

### 2.3. Nutritional Supplementation

The exact composition of and nutrition information for the MIS and PLA capsules is provided in Table 1. The supplement provided an energetic value of ∼10 kcal per capsule, in a capsule weight of ~0.6 g. Participants were asked to consume 2 capsules ~30 min prior to the consumption of a meal, three times per day (i.e., breakfast, lunch, and dinner). Therefore, participants were instructed to consume a total of 6 capsules of the study supplement per day (7 d/wk^−1^) throughout the 12 wk intervention period. The MIS provided ~200 mg of naturally sourced caffeine (primarily from green coffee bean extract) per day. Participants were given a 4 wk supply during monthly visits and maintained the used package and turned it in at the check-ins as a marker of study compliance. The DER (Drug Extract Ratio) and extraction solvents for plant-based extracts are found in Supplemental Materials Table S1.

### 2.4. Anthropometric Measurements and Body Composition

Participants were weighed at baseline and at each follow-up study visit. Weight was measured using a Health-O-Meter 2600KL Digital Wheelchair Scale (Pelstar, McCook, IL, USA), and height was measured using a stadiometer (Perspective Enterprises, Portage, MI, USA). Waist and measurements for the calculation of WHR [54,55] were performed with a Gulick tape measure. A horizontal measure was obtained directly above the iliac crest and for hip circumference measurement, the participants stood erect with feet together, and a horizontal measure was taken at the maximal circumference of the buttocks [60]. Fat-free mass (FFM) and fat mass (FM) were assessed using a DXA scan (GE Lunar Prodigy, Madison, WI, USA) and a software program (encore Version 9.15.010). Whole body composition assessed via DXA was validated in vivo [61,62,63]. A single investigator performed regional body compartment analysis prior to unblinding. Participants underwent all anthropometric measurements and DXA scans in a clean hospital gown with the same undergarments for every visit.

### 2.5. Measurement of Blood Pressure

Participants were fitted with a FlexiPort™ reusable blood pressure cuff (WelchAllyn, Inc., Okumoto, NY, USA) to measure blood pressure while the participant was relaxed and seated for 15 min. Measurements were obtained with an automated blood pressure machine (Spot Vital Signs Device, WelchAllyn).

### 2.6. Venous Blood Sampling and Analysis

Blood was collected in the morning following an overnight 10 h fast (no food or caffeine) and the participants were instructed to consume 250 mL of water prior to arrival. Blood was taken from the antecubital vein and drawn into evacuated tubes with heparin for plasma collection, ethylenediaminetetraacetic acid (EDTA) for plasma collection, and non-treated tubes were used to collect serum. Serum and plasma samples were immediately taken to the Core Laboratory at McMaster University Medical Centre for the analysis of the following panel of tests for general blood biochemistry: complete blood count (CBC), gamma-glutamyl transferase (GGT), bilirubin, alanine aminotransferase (ALT), creatine kinase (CK), creatinine, and C-reactive protein (CRP). Markers of dyslipidemia (i.e., triglycerides, total cholesterol, LDL cholesterol, and HDL cholesterol) and blood sugar control (i.e., glucose, insulin) were measured. Homeostatic Model Assessment of Insulin Resistance (HOMA-IR) was calculated as the equation [fasting insulin (U/L) × fasting glucose (nmol/L)/22.5] [64]. If available, the remaining samples were stored at −80 °C for subsequent analysis. To verify the ALT findings, aspartate transferase (AST) levels were also assessed via Core Laboratory during secondary analysis. For exploratory analysis, plasma samples were batch-analyzed via enzyme-linked immunosorbent assay (ELISA) for leptin (Quantikine DLP00, R&D Systems, Minneapolis, MN, USA), IL-6 (Quantikine D6050, R&D Systems), CoQ10 (MBS165643, BioSourceLLC, San Diego, CA, USA), FGF21 (ab222506, Abcam, Cambridge, UK), and GDF15 (Quantikine DGD150, R&D Systems) per kit instructions, respectively. All investigators and laboratory technicians were unaware of the participants’ group assignment during the initial downstream analysis.

### 2.7. Isolation of Extracellular Vesicles

Exactly 400 µL of serum was initially subjected to two consecutive centrifugation spins at 2000× *g* and 20,000× *g* (Beckman Coulter Optima MAX-TL, Pasadena, CA, USA) for 30 min each at 4 °C to remove any large EVs. Following differential centrifugation, exactly 300 µL of supernatant containing small EVs was carefully retained and subjected to 16 h of ultracentrifugation (Beckman Coulter Optima MAX-TL, CA, USA) at a speed of 120,000× *g* at 4 °C. A total of 250 µL of supernatant was carefully removed, whereas the fluid pellet was washed with 250 µL of 1X Dulbecco’s phosphate-buffered saline (D8537, Sigma-Aldrich, Burlington, MA, USA) and briefly sonicated to ensure proper resuspension of the fluid pellet. The solution was further spun at a similar speed for 60 min where a final volume of 100 µL of fluid pellet was retained and used for miRNA extraction.

### 2.8. miRNA Extraction and RT-PCR

The MagMAX™ *mirVana*™ *Total RNA* Isolation Kit (A27828; Thermo Scientific, Waltham, MA, USA) was utilized as per the manufacturer’s instructions. Briefly, 100 µL of EV isolates were plated onto extraction plates where magnetic beads aided in the isolation of 50 μL of total RNA. Prior to the extraction, *cel-miR-54*-3p (custom oligo; Thermo Fischer Scientific) spike-in was added to each sample at a concentration of 1 × 10^−9^ M. Two µL of eluted RNA was then used as input to synthesize cDNA using TaqMan™ Advanced miRNA cDNA Synthesis Kit (A28007; Thermo Scientific). The expression of the miRNAs of interest (Supplemental Materials Table S2) was then measured using TaqMan™ Fast Advanced Master Mix (4444558; Thermo Fisher Scientific) and the CFX Connect Real-Time PCR Detection System (BioRad, Hercules, CA, USA). The *cel-54-3p* was used as a reference gene for normalization and fold change between baseline (Day 0) and 12 wk period (end of intervention) was calculated using the 2^−ΔΔ*C*^_T_ method [65].

### 2.9. Metabolic Measures and Resting Metabolic Rate (RMR)

Participants were placed in a supine position while connected to a metabolic cart with an online gas collection system (Moxus Modular Oxygen Uptake System, AEI Technologies, Pittsburgh, PA, USA), and the system acquired oxygen consumption (VO_2_) and carbon dioxide (CO_2_) production data, which was recorded after 30 min of rest. For the duration of the test, the participants were instructed to remain relaxed without falling asleep. From this data, the abbreviated Weir equation was used to calculate RMR [RMR = 1440 × (VO_2_ × 3.941) + (VCO_2_ × 1.11)] [66,67]. The RMR was also estimated (eRMR) using the standard Mifflin-St. Jeor equation [68], which was validated in overweight/obese populations [69,70]. For females, RMR was calculated as the equation [RMR = 9.99 × weight + 6.25 × height − 4.92 × age − 161]. For males, RMR was calculated as the equation [RMR = 9.99 × weight + 6.25 × height − 4.92 × age + 5]).

### 2.10. Maximal Voluntary VO_2peak_ Fitness Test

Participants completed a double-leg incremental peak oxygen uptake (VO_2peak_) test on a cycle ergometer (Lode, Groningen, The Netherlands). A metabolic cart with an online gas collection system (Moxus Modular Oxygen Uptake System, AEI Technologies, Pittsburgh, PA, USA) acquired oxygen consumption (VO_2_) and carbon dioxide (CO_2_) production data. The test began with a 2 min warm-up at 30 watts (W), after which the power was increased by 30 W every minute until volitional exhaustion or when the pedal cadence fell below 55 rpm. Heart rate was monitored continuously throughout the test via telemetry with a heart rate monitor (Polar A3, Lake Success, NY, USA). VO_2peak_ was defined as the highest oxygen consumption achieved over a 30 s period. Maximal workload (W_max_) was the highest power output achieved during the test. After the completion of the test, the participant performed a 3 min active recovery pedaling against 30 W.

### 2.11. Maximal Voluntary Handgrip Assessment

Handgrip strength was measured using an isometric dynamometer (JAMAR^®^, Sammons, Bolingbrook, IL, USA). The grip width was adjusted to hand size and with the arm flexed at 90° while seated. The participant performed three separate maximal 3 s trials with a 1 min rest period between trials, and the mean value was taken.

### 2.12. Questionnaires

The RAND 36-Item Health Survey Version 1.0 is used extensively as a survey instrument for assessing participant health-related quality of life (HRQOL). We examined scales pertaining to physical functioning and role limitations due to health problems instead of the total score [71]. These domains were scored on a 0 to 100 range such that a higher score was representative of a higher quality of life [72]. These scales are validated [73] and have been utilized in the context of weight loss [74].

### 2.13. Dietary Intake

Three-day food records (recording 2 weekdays and 1 weekend day) were collected and analyzed using the MyFitnessPal smartphone app (MyFitnessPal, Under Armour, Baltimore, MD, USA.) and website that tracks diet and exercise. If participants were not comfortable with utilizing the smartphone application, they were provided with a paper diet log. Participants were given instructions on how to record their intake of any food and beverages. Diet logs were completed at periods before the participant began the study protocol, at the study midpoint (5 wk), and final timepoint (12 wk).

### 2.14. Activity Tracking

Study participants were provided with a pedometer (Omron HJ-321, Omron, Kyoto, Japan) to record their daily step counts for three separate 7 d periods within the study to determine average daily step count. Daily step count for a 7 d period was recorded at periods before the participant began the study protocol, at the study midpoint (5 wk), and final timepoint (12 wk).

### 2.15. Sample Size Calculation

The MIS was comprised of 7 separate ingredients, with aspects featuring a potential varied or synergistic [52] impact on body composition. Sample sizes were calculated based on a detection difference in fat mass (kg) loss of 4.01 kg (sample standard deviation: 4.5 kg) modified from observations of the effect of oral ingestion of forskolin (derived from *Coleus forskohlii*) on body composition [46]. The sample size was calculated with a power of 80% and a significance level of 0.05 (2-tailed), which yielded an estimate of approximately 20 participants per group. To account for an ~25% drop-out rate [75] observed in similar trials using nutritional supplementation and inherent variability in weight management when participants performed some levels of baseline physical activity [76], at least 25 participants were recruited.

### 2.16. Statistical Analysis

Post-intervention differences between treatment arms in outcome variables were compared using a one-way analysis of covariance (ANCOVA), implementing the corresponding pre-intervention variables as covariates. If data were not normally distributed, logarithmic transformations were performed. Endpoints that were intractably non-normal were assessed using the Mann–Whitney U test. Within-group analysis was performed utilizing a paired *t*-test or, in the case of intractable non-normality, the Wilcoxon sign rank test. Exclusively for co-primary outcomes (i.e., weight and fat mass), statistical analysis was performed using a modified intention-to-treat (ITT) population, wherein all participants who were randomized to receive PLA or MIS, and on whom any post-randomization information prior to dropout or completion was available. This is in accordance with recently suggested practices for randomized weight loss trials [77]. In this case, all missing values were imputed using the last observation carried forward (LCOF) method. Following the co-primary outcome analysis, statistical analysis was performed on all anthropometry, clinical biochemistry, and lifestyle data (e.g., step count and diet logs) exclusively for participants who completed the intervention. Statistical significance was set at α < 0.05. SPSS (version 28; SPSS, Inc., Chicago, IL, USA) was used for all statistical analyses. During the intervention period, the SARS-CoV-2 virus pandemic impacted Canada. Coronavirus disease (COVID-19) is an infectious disease caused by the SARS-CoV-2 virus. At the time (April 2020), the infection vector had not been well described, and to reduce close contact and/or infectious aerosol particulates transported between research staff and participants in the trial, the final participant cohort (*n* = 5 PLA, *n* = 7 MIS) did not have a final blood draw/analysis, RMR, resting HR, grip strength, resting heart rate, VO_2peak,_ SBP, or DBP measurements taken at the endpoint (i.e., 12 wk mark). The LCOF method was not used to replace data for these measurements in this cohort.

## 3. Results

### 3.1. Study Information and Compliance

A total of 65 participants were randomized: 55 completed the study, five dropped out (*n* = 3, and *n* = 2 dropouts in the PLA and MIS groups, respectively), and three were lost to follow-up (*n* = 3 MIS). One participant was removed from the study due to initiating a medication known to alter protein metabolism during the intervention period (*n* = 1 MIS), and one participant was removed due to non-compliance with study visits (*n* = 1 MIS). Of the participants who withdrew from the study or were lost to follow-up following randomization, *n* = 2 withdrew prior to Visit 2, *n* = 3 withdrew prior to Visit 3, and *n* = 3 withdrew prior to Visit 4 (endpoint). The reasons for participant withdrawal or removal are listed in Supplemental Figure S1. Treatment arm compliance was assessed by pill counting from the returned supplement package per visit (monthly). Supplement consumption compliance over the duration of the intervention was 82.7 ± 4.6% and 85.4 ± 3.1% in the PLA and MIS groups, respectively. No significant adverse events directly related to the treatment were recorded or shared by the participants. Three participants reported mild gastric reflux immediately after the intervention (*n* = 1 PLA; *n* = 2 MIS). These symptoms were not reported following the first week (subsiding by Visit 2; ~2 weeks following initiation of intervention), and no participants withdrew from the study due to events or concerns related to consuming the PLA or MIS.

### 3.2. Baseline Characteristics

Fifty-five participants (23 men, 32 women) with a mean age of 25.9 ± 1.1 y (mean ± SEM) completed the study. The participants were overweight to obese, with a mean BMI of 30.5 ± 0.6 kg/m^2^. Pre-intervention, there were no significant differences between treatment arms in weight, BMI, total fat mass, total fat-free mass, or waist-to-height ratio (*p* ≥ 0.05, Table 2).

### 3.3. Co-Primary Outcomes Utilizing Modified ITT

For the examination of weight and fat mass (co-primary outcomes) exclusively, we utilized a modified ITT analysis. After adjustment for pre-intervention body weight, there was a statistically significant difference in post-intervention body weight between the PLA and MIS interventions, *F*(1, 52) = 79.090, *p* = 0.001, partial η^2^ = 0.183. In the PLA group, there was a significant increase in body weight at the endpoint compared to the baseline (*p* = 0.033). In the MIS group, there was a significant decrease at the endpoint compared to the baseline (*p* < 0.001). After adjustment for pre-intervention fat mass, there was a statistically significant difference in post-intervention fat mass between the PLA and MIS interventions: *F*(1, 52) = 31.871, *p* = 0.031, and partial η^2^ = 0.087. During the intervention, there was a significant within-group difference from baseline (Day 0) to endpoint in both PLA (increased, *p* = 0.049) and MIS (decreased, *p* = 0.034).

### 3.4. Anthropometry and Body Composition

**Figure 1:**
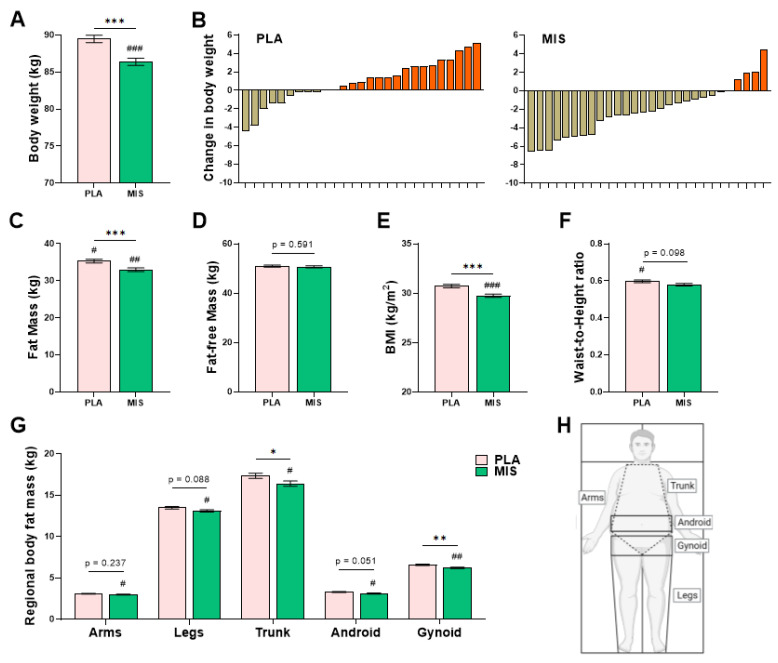
Characterization of anthropometric changes following 12 wks of a multi-ingredient supplement (MIS, *n* = 28, green bars) compared to placebo (PLA, *n* = 27, pink bars). Post-intervention differences between treatment arms were compared using one-way ANCOVA, implementing the corresponding pre-intervention variables as covariates. Post-intervention data are presented as adjusted means ± SEs. Asterisks (*) denote significant (*p* < 0.05) between-group differences versus PLA, *p* ≥ 0.05, *; *p* ≤ 0.05, **; *p* ≤ 0.01, ***; *p* ≤ 0.001. Number signs (#) denote significant within-group differences relative to Day 0 (baseline), assessed via paired *t*-test, #; *p* ≤ 0.05, ##; *p* ≤ 0.01, ###; *p* ≤ 0.001. (**A**) Post-intervention weight (kg). (**B**) Individual change (Δ) in body weight (kg) following 12 wk intervention in the PLA and MIS group. (**C**) Post-intervention DXA-derived fat mass (kg). (**D**) Post-intervention DXA-derived fat-free mass (kg). (**E**) Post-intervention BMI (kg/m^2^). (**F**) Post-intervention waist-to-height ratio (a.u.). (**G**) Post-intervention DXA-derived regional body fat mass (kg). (**H**) Representative image of regional body fat mass demarcations.

After adjustment for pre-intervention body weight, there was a statistically significant difference in post-intervention weight between the treatment arms (*p* ≤ 0.001, Figure 1A). Participants in the MIS treatment arm significantly decreased in weight from baseline (*p* ≤ 0.01, Δ −2.2 ± 2.8 kg, mean ± SD), and those in the PLA treatment arm tended to increase in weight from baseline (*p* = 0.052, Δ 0.9 ± 2.4 kg). All individual participant changes in weight over the period of the intervention are shown for the PLA and MIS treatment arms, respectively (Figure 1B).

After adjustment for pre-intervention fat mass, there was a significant difference in post-intervention fat mass between the treatment arms (*p* ≤ 0.001, Figure 1C). Following the intervention period, participants in the MIS arm had significantly decreased fat mass (*p* ≤ 0.01, Δ −1.4 ± 2.5 kg), whereas those in the PLA group tended to have increased fat mass (*p* = 0.052, Δ 0.9 ± 2.7 kg).

There was no significant difference in the post-intervention total fat-free mass between the treatment arms (*p* = 0.591, Figure 1E), adjusted for pre-intervention data. No changes were observed in either treatment arms (*p* ≥ 0.05, Table 3).

The ratio of fat-free mass (kg) to fat mass (kg), termed herein the Body Composition Index (BCI), was significantly different between the intervention arms (*p* = 0.034, Table 3) following the intervention. The BCI of the MIS treatment arm was significantly increased compared to baseline (*p* < 0.05, Δ 0.1 ± 0.2 a.u.), corresponding with the relative loss of fat mass and the relative preservation of fat-free mass.

The BMI adjusted for pre-intervention values was significantly different between the treatment arms (*p* ≤ 0.001, Figure 1F), with the MIS group having significantly decreased BMI from baseline (*p* ≤ 0.001, Δ −0.7 ± 0.9 kg/m^2^), but there was no difference in the PLA group (*p* = 0.086, Δ 0.3 ± 0.8 kg/m^2^).

Following adjustment for pre-intervention data, there was no significant difference in the post-intervention waist-to-height ratio between the treatment arms (*p* = 0.098, Figure 1G), with participants in the PLA treatment arm significantly increasing from baseline (*p* < 0.05, Δ 0.02 ± 0.04 a.u).

There were significant differences detected between the treatment arms in the post-intervention regional fat mass in the trunk and gynoid regions (*p* = 0.039 and *p* = 0.002, respectively), adjusted for pre-intervention values (Figure 1G). In the MIS group, the trunk (*p* = 0.014, Δ −0.6 ± 1.3 kg) and the gynoid (*p* ≤ 0.001, Δ −0.3 ± 0.4 kg) regional fat masses were significantly lower following the intervention period. Following adjustment for the respective baseline variables, there were no significant differences in post-intervention arm fat mass (*p* = 0.237), leg fat mass (*p* = 0.088), or android fat mass (*p* = 0.051) between the treatment arms (Table 3). Following the intervention, participants in the MIS treatment arm had significantly reduced arm fat mass (*p* < 0.05, Δ −0.3 ± 1.3 kg), leg fat mass (*p* < 0.05, Δ −0.4 ± 0.8 kg), and android fat mass (*p* < 0.05, Δ −0.1 ± 0.2 kg) respectively. At baseline, there were no significant differences in regional fat mass (kg) between the treatment arms (*p* ≥ 0.05).

### 3.5. Clinical Biochemistry

Following adjustment for pre-intervention ALT, there was a significant difference in post-intervention ALT between the treatment arms (*p* = 0.007, Table 4). Following the intervention, participants in the MIS group showed a significant decrease from baseline (*p* < 0.05, Δ −7.0 ± 13.2 U/L). Correspondingly, significant post-intervention differences were detected in AST between treatment arms (*p* = 0.017, Table 4), adjusted for pre-intervention data, with the MIS group significantly decreasing from baseline (*p* < 0.05, Δ −4.5 ± 8.7 U/L).

Following adjustment for the respective baseline variables, there were no significant differences in post-intervention serum creatinine (*p* = 0.089, Table 4) between the treatment arms. Following the intervention, participants in the MIS treatment arm had significantly increased endpoint creatinine levels (*p* < 0.05, Δ 4.0 ± 5.6 µmol/L) compared to baseline, respectively.

There were no significant differences between the treatment arms in GGT, CRP, or bilirubin (*p* ≥ 0.05, Table 4) between the two treatment arms, adjusted for their respective pre-intervention values, nor were there any significant differences within the treatment arm at the endpoint compared to baseline (*p* ≥ 0.05, Table 4).

### 3.6. Markers of Dyslipidemia and Glucose Metabolism

There were no significant differences between the treatment arms with respect to total cholesterol, low-density lipoprotein cholesterol (LDL), high-density lipoprotein cholesterol (HDL), and triglycerides (*p* ≥ 0.05, Table 4) between the two treatment arms, adjusted for their respective pre-intervention values. No significant post-intervention differences were detected in fasting glucose, insulin, or derived HOMA-IR, adjusted for pre-intervention values (*p* ≥ 0.05, Table 4). At baseline, there were no significant differences in circulating lipid markers or glucose metabolism outcomes between (*p* ≥ 0.05) or within (*p* ≥ 0.05) the treatment arms.

### 3.7. Hematology

After adjustment for pre-intervention circulating eosinophil cell population, there was a statistically significant difference in post-intervention data between the treatment arms (*p* ≤ 0.01, Table 5), with the PLA group significantly increasing from baseline (*p* < 0.05, Δ 0.03 ± 0.1 × 10^9^/L). There were no significant differences between the treatment arms in hemoglobin (Hb), hematocrit (HCT), mean corpuscular volume (MCV), mean corpuscular hemoglobin (MCH), mean corpuscular hemoglobin concentration (MCHC), mean platelet volume (MPV), or red cell distribution width (RDW) after adjustment for corresponding pre-intervention data (*p* ≥ 0.05, Table 5). Cell quantification of leukocytes, erythrocytes, platelets, neutrophils, lymphocytes, monocytes, and basophils did not differ post-intervention between the two treatment arms, adjusted for their corresponding pre-intervention values (*p* ≥ 0.05, Table 5). These hematology outcomes were unaltered following the intervention in both treatment arms (*p* ≥ 0.05).

### 3.8. Indices of Physical Health, Metabolism and Function

No statistically significant differences were found between the two treatment arms in post-intervention systolic blood pressure, diastolic blood pressure, resting heart rate, or bone mineral density, adjusted for the corresponding pre-intervention variable data (Table 6). Following adjustment for pre-intervention relative grip strength, there was a significant post-intervention difference between the treatment arms (*p* = 0.002, Table 6). Following the intervention, participants in the MIS group significantly increased from baseline (*p* < 0.05, Δ 0.07 ± 0.06 kg/kg bw^−1^), whereas participants in the PLA group significantly decreased from baseline (*p* < 0.05, Δ 0.02 ± 0.04 kg/kg bw^−1^), in line with changes in total body weight observed following the intervention period. Relative VO_2peak_ was not significantly different post-intervention between the treatment arms after correcting for baseline data (*p* ≥ 0.05, Table 6). The resting metabolic rate (RMR), as measured by gas exchange data *in vivo*, was not significantly different post-intervention between the treatment arms after correcting for baseline data (*p* ≥ 0.05, Table 6).

### 3.9. Diet, Activity Level, and Self-Reported Quality of Life

At baseline, there was no significant difference between the treatment arms in terms of energy intake, caffeine consumption, and 7-day pedometer-derived average daily step count (*p* ≥ 0.05). Energy intake derived from the 3-d dietary recall log (kcal/d) was not different between the treatment arms (*p* = 0.856) after adjustment for pre-intervention energy intake. The adjusted mean of energy intake was 1802.1 ± 98.9 kcal/d^−1^ in the PLA arm compared to 1761.8 ± 97.1 kcal/d^−1^ in the MIS arm. There were no significant differences in energy intake per day at the endpoint compared to baseline within either group (*p* ≥ 0.05). Caffeine consumption was not different between the treatment arms (*p* = 0.569) after adjustment for the corresponding pre-intervention data. The pre-intervention adjusted mean dietary caffeine consumption was 66.4 ± 13.9 mg/d^−1^ in the PLA arm compared to 67.1 ± 13.4 mg/d^−1^ in the MIS arm. The MIS group also consumed an additional ~200 mg/d^−1^ of caffeine contained as part of the MIS formulation (primarily from GCB extract). There were no significant differences in caffeine intake at the endpoint compared to baseline within either group (*p* ≥ 0.05). Habitual alcohol consumption was self-reported at the onset of the intervention. In the PLA treatment arm, 51.9% (14/27) never consumed alcohol, 22.2% (6/27) occasionally consumed alcohol, 18.5% (5/27) consumed alcohol on a weekly basis (less than 5d per week), and 7.4% (2/27) consumed one unit of alcohol daily. In the MIS treatment arm, similar proportions were observed with 39.3% (11/28) never having consumed alcohol, and 28.6% (8/28), 21.4% (6/28), and 10.7% (3/28) consuming occasional, weekly, or daily alcohol, respectively. The step count was not different between the treatment arms (*p* = 0.320) following adjustment for pre-intervention data. The adjusted mean for step count was 6152.7 ± 822.7 steps/d^−1^ in the PLA arm compared to 6156.5 ± 807.4 steps/d^−1^ in the MIS arm. There were no significant differences in the daily step count at the endpoint compared to the baseline within either MIS or PLA group (*p* ≥ 0.05). The SF-36 scales associated with physical functioning were not significantly different between treatment arms at baseline (PLA; 90.6 ± 3.7, MIS; 93.0 ± 2.3) and were not different between groups (*p* = 0.262) or within groups (*p* ≥ 0.05) following the intervention period. The scores associated with role limitations due to physical health problems were not significantly different at baseline between the PLA (90.4 ± 4.1) and MIS (95.5 ± 2.8) and were not significantly different between the treatment arms (*p* = 0.429) or within groups (*p* ≥ 0.05).

### 3.10. Molecular Signalling Factors and Antioxidant Capacity

**Figure 2:**
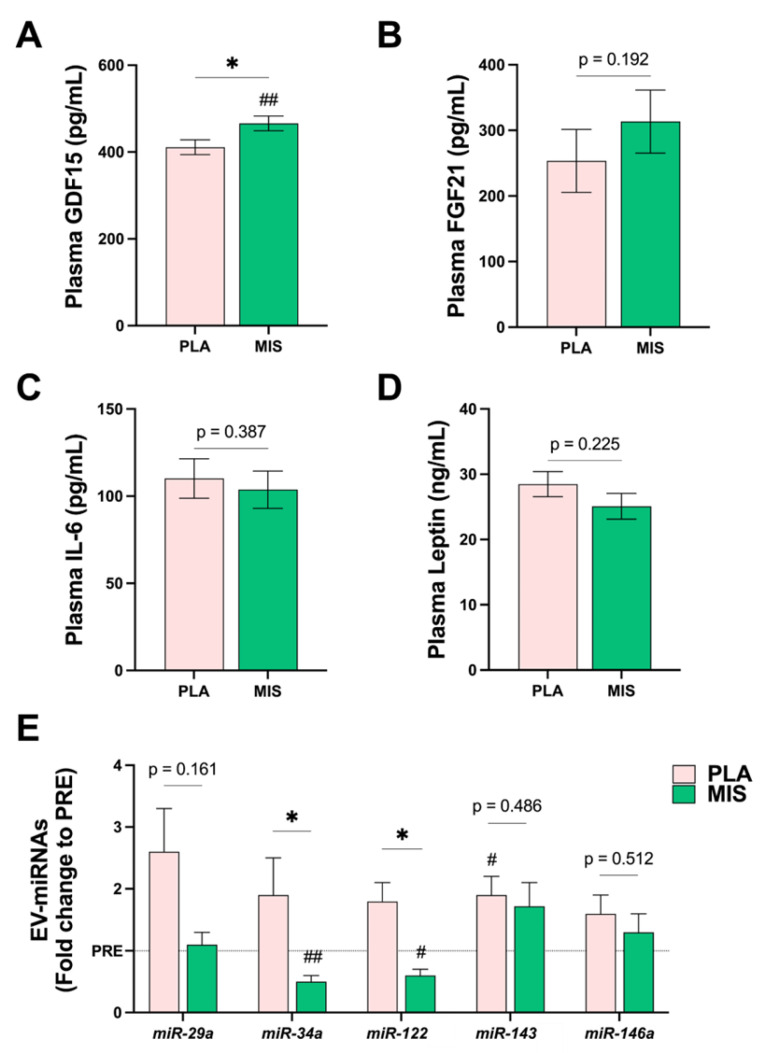
Characterization of plasma and circulating molecular signaling factors following 12 wks of a multi-ingredient supplement (MIS, green bars) compared to placebo (PLA, pink bars). (**A**) Post-intervention plasma GDF15 (pg/mL). Post-intervention differences between treatment arms were compared using one-way ANCOVA, implementing the corresponding pre-intervention variables as covariates. Within-group comparisons were made using a paired *t*-test. (**B**) Post-intervention plasma FGF21 (pg/mL). (**C**) Post-intervention plasma IL-6 (pg/mL). (**D**) Post-intervention plasma leptin (ng/mL). (**E**) Extracellular vesicle (EV)-associated miRNA species shown as fold change from Day 0 (baseline), *n* = 15 per group. miRNAs are normalized to exogenous miRNA, *cel-miR-54* added prior to RNA isolation. Between-group comparisons were made using the Mann–Whitney U test. Within-group comparisons were made using the non-parametric sign rank test. Asterisks (*) denote significant (*p* < 0.05) between-group differences versus PLA, *p* ≥ 0.05, *; *p* < 0.05. Number signs (#) denote significant within-group differences relative to Day 0 (baseline), #; *p* < 0.05, ##; *p* ≤ 0.01. Post-intervention data are presented as adjusted means ± SEs. GDF15, Growth differentiation factor 15; FGF21, fibroblast growth factor 21; IL-6, interleukin 6; miRNA, microRNA.

Post-intervention growth differentiation factor 15 (GDF15) was significantly different between the two treatment arms, following adjustment for pre-intervention data (*p* = 0.028, Figure 2A). Circulating GDF15 protein content was significantly increased in the MIS group compared to baseline (*p* ≤ 0.01, Δ 42.6 ± 53.8 pg/mL). There were no significance differences between the treatment arms in circulating fibroblast growth factor 21 (FGF21, Figure 2B), interleukin 6 (IL-6, Figure 2C), or leptin (*p* ≥ 0.05, Figure 2D) between the two treatment arms, adjusted for their respective pre-intervention values (Table 7).

The circulating EV-associated miRNA species miR-34a (*p* = 0.045) and miR-122 (*p* = 0.016) were significantly different between the treatment arms post-intervention (Figure 2E). EV miRNA miR-34a (~0.5-fold) and miR-122 expression (~0.6-fold) were significantly downregulated in the MIS treatment arm following the intervention (*p* < 0.05, Figure 2E). In the PLA group, miR-34a (*p* = 0.570) and miR-122 expression (*p* = 0.061) were not statistically different from the baseline (Figure 2E). While there were no significant differences between the treatment arms post-intervention (*p* ≥ 0.05), EV miRNA-143 was significantly upregulated in the PLA group (*p* < 0.05, Figure 2E, ~1.9-fold). miR-29a and miR-146a were not statistically different between treatment arms following the intervention (*p* ≥ 0.05, Figure 2E) and did not change over the intervention period within-the group.

**Figure 3:**
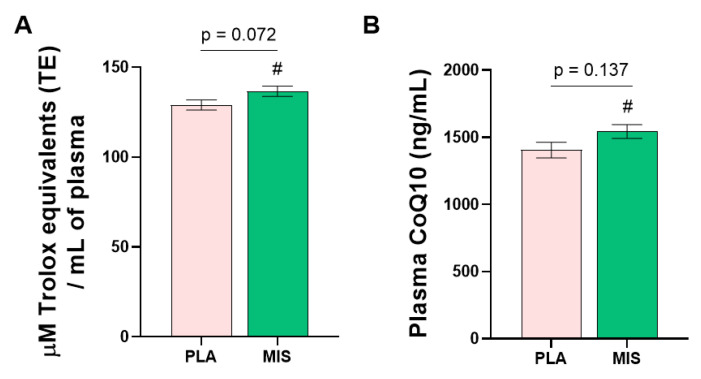
Characterization of plasma antioxidant content and capacity following 12 wks of a multi-ingredient supplement (MIS, green bars) compared to placebo (PLA, pink bars). (**A**) Post-intervention plasma Trolox equivalents. Post-intervention variables were compared using one-way ANCOVA, with corresponding baseline variables as covariates. Within-group differences relative to Day 0 (baseline) were assessed via paired *t*-test (**B**) Post-intervention plasma CoQ10 content. Between-group comparisons were made using the Mann–Whitney U test. Within-group comparisons were made using the non-parametric sign rank test. Number signs (#) denote significant within-group differences relative to Day 0 (baseline), #; *p* < 0.05. Post-intervention data are presented as adjusted means ± SEs. CoQ10; coenzyme Q10.

Oxygen Radical Absorbance Capacity (ORAC) within the blood plasma was not significantly different the between treatment arms (*p* = 0.072, Figure 3A) adjusted for pre-intervention values. The MIS group significantly increased plasma ORAC from baseline compared to the endpoint (*p* < 0.05, Δ 6.2 ± 12.8 µM trolox equivalents/mL). Circulating CoQ10 was not significantly different between the intervention arms (*p* = 0.137, Figure 3B) after adjustment for pre-intervention data. Plasma CoQ10 levels were significantly increased in the MIS arm after treatment (*p* < 0.05, Δ 0.09 ± 0.17 µg/mL).

## 4. Discussion

We report that the daily consumption of a multi-ingredient supplement (MIS) designed to facilitate weight loss and mitochondrial function [52] resulted in significant loss of body weight, being driven predominately by a reduction in fat mass with the preservation of fat-free mass. In addition, other clinically significant outcomes, such as BMI and WHR, were significantly reduced following a 12 week intervention period in the MIS treatment arm. These changes occurred in the absence of major dietary changes and/or changes in step count (as a marker of physical activity) by participants during the experimental period, suggesting that the observed changes were driven by MIS. We observed a significant improvement in blood biochemical outcomes, including AST and ALT, and a reduction in extracellular vesicle-derived miRNAs associated with obesity, liver dysfunction, and inflammation. Taken together, our findings suggest that a seven-component, multi-ingredient nutritional supplement (MIS) results in significant weight and total fat mass loss in comparison with PLA in overweight and/or obese men and women.

Amongst an overweight population, there is a significantly lower mortality risk for individuals who are classified as overweight (BMI of 25.0–29.9 kg/m^2^), as compared to a greater mortality risk for individuals who have a BMI range of 35.0–35.9 [7,78,79]. Similarly, higher body fat content has been shown to be associated with a higher risk of mortality [80]. To counteract the insidious risk of developing co-morbidities associated with being overweight and/or a progression to obesity, a significant weight loss of 5–10% is suggested by health care practitioners [81]. Concomitant with a clinically relevant reduction in weight of ~2.5% (−2.2 kg), we report a fat mass reduction of ~4% in the MIS treatment arm. Despite this significant reduction in weight and fat mass, we observe no overall reduction in FFM or bone mineral density. These findings highlight the potential therapeutic value of nutritional MIS, given the loss of FFM as a part of the total weight loss observed with other weight management strategies. Indeed, bariatric surgery leads to significant, if not massive, weight and fat loss, as well as a significant loss (~30%) of muscle and bone mass [82]. Recent pharmacological interventions, such as glucagon-like peptide-1 receptor agonists and sodium-glucose co-transporter-2 inhibitors (SGLT2i), can facilitate clinically relevant weight loss in individuals with type 2 diabetes [83,84,85]. There is evidence to suggest that both semaglutide (i.e., an approved GLP-1RA) and canagliflozin (i.e., an approved SGLT-2i) can reduce total FFM [86,87], though whether this has a significant impact on the proportion of lean mass to total mass is still being debated in the literature [87,88,89]. Even less robust means of weight loss such as dietary energy restriction can present challenges for the preservation of lean body mass. In individuals who are overweight or obese, the loss of FFM can be ~20–30% of the total observed weight loss [90,91,92]. From a mechanistic perspective, Hector and colleagues observed reductions in measured integrated muscle protein synthesis (MPS) assessed via muscle biopsy in response to an energy deficit [93] in overweight adult men. Previous work suggested that post-absorptive muscle protein breakdown (MPB) increased by ~60% following 10 days of 20% energy restriction [94], underscoring the findings that extensive dietary energy restriction can lead to a reduction in body mass, with ~25% of FFM/muscle [91]. Given that skeletal muscle features high metabolic activity and is the largest component of FFM, a loss of muscle can lead to a reduction in mobility and/or metabolic health. Thus, care must be taken to preserve the muscle during weight loss [95]. Collectively, our data suggest that MIS can facilitate weight loss while promoting the maintenance of FFM, a clinically relevant feature of therapeutic strategies to combat obesity and obesity-related comorbidities. Given that the MIS used in the current study predominantly acts via a different mechanism compared to GLP-1RA medications (browning of white adipose tissue vs. appetite suppression, respectively), future studies should evaluate the potential for weight loss synergy and the potential for FFM preservation with concurrent use.

By design, it is not possible to isolate which individual aspects of the seven-component MIS contributed to our observations; each has been shown to independently facilitate weight loss and/or improve mitochondrial function. We have previously reported that 5-, 7-, and 10-component MIS significantly reduced body weight and fat mass, and improved markers of in vivo metabolism in a murine model [52]. Notably, green tea catechins (particularly epigallocatechin-3-gallate) [96] have been well-documented to facilitate a reduction in body weight and body fat tissue in humans over a similar experimental period [97,98,99,100,101]. Mechanistically, green tea (and/or its extracts) may alter SNS activation and lipolysis [102,103], downregulate fatty acid synthase [104], and induce thermogenesis [105]. Similarly, both forskolin [46,106] and green coffee beans [48,107] have been shown to have beneficial effects on weight loss and body re-composition in favor of lower fat mass. Forskolin has been shown in vitro and in animal models to activate cyclic AMP, stimulate fat oxidation, directly activate hormone-sensitive lipase, and reduce body weight [108], and has been recently shown to improve insulin resistance in obese adults [109]. Chlorogenic acid (CA), a bioactive component of coffee beans that is greater in green bean coffee compared to the roasted counterpart [110], has been shown to inhibit hepatic glucose-6-phosphatase, the rate-limiting enzyme involved in gluconeogenesis [111], and promote the reduction in hepatic triglycerides in conjunction with caffeine in obese rats [112].

The composition of MIS was designed to also include bioactive compounds that could facilitate mitochondrial function, given the established relationship between mitochondrial dysfunction and the pathogenesis of obesity. Increased consumption of food can lead to increased circulating lipids and carbohydrates, providing an ‘over-supply’ of energy substrates in metabolic tissues, which in turn stimulates the production of ROS [22]. We have previously utilized these aspects in a clinical trial in patients with mitochondrial disease, ultimately improving mitochondrial function (i.e., lower lactate) and reducing oxidative stress [113]. Vitamin E is an important lipid-soluble antioxidant [114] and may decrease fatty liver development [38,115]. Some clinical trials have observed that α-facilitates weight loss in humans [116]; whereas, others have observed no significant effect of α-alone on weight loss. However, α-is unequivocally important for mitochondrial function [117] and is a significant scavenger of free radicals [118]. Plasma antioxidant capacity intake has been shown to be correlated with the dietary intake of antioxidants [119]; therefore, we confirmed our hypothesis that antioxidant capacity in plasma (as measured by oxygen radical absorbance capacity) would significantly increase in the MIS treatment arm over time. In line with the provision of CoQ10 as an aspect of the 7-component MIS, we observed that participants in the MIS treatment arm had a trend for an elevated CoQ10 following the supplementation period. Absorbed CoQ10, when provided via nutritional supplementation, has a half-life of ~34 h, with T_max_ occurring at ~6 hrs [120]. Given that blood collection was performed ~10–12 h following supplementation (i.e., the last meal), it stands to reason that CoQ10 and antioxidant capacity in the blood would still be elevated if measured earlier following ingestion. Using a pre-clinical model of obesity, we have previously reported significant improvement in body composition (i.e., lower relative fat and higher relative muscle mass) in mice fed a high-fat diet using components including weight loss-inducing agents with concomitant inclusion of ingredients known to facilitate mitochondrial function [52]. Irrespective of the individual contributions of the MIS components, pre-clinical evaluation of the MIS ingredients did lead to an increase in mitochondria in murine white adipose tissue. Taken together, the combined approach utilizing mitochondrial- and fat-loss-targeting ingredients may be uniquely beneficial.

Fatty liver disease, when the triglyceride content of the liver organ weight surpasses 5%, is the most common liver disease associated with individuals who are obese [121]. When independent of alcohol consumption, this disease can be classified as non-alcoholic fatty liver disease (NAFLD). Our examination of alcohol consumption in both the PLA and the MIS treatment arms revealed no significant differences between the groups and a relatively low consumption across participants in the study. The prevalence of NAFLD and the subsequent progression into non-alcoholic steatohepatitis (NASH) is linked to a large degree of obesity, with 65% of individuals presenting with grade I-II obesity (BMI = 30–39.9 kg/m^2^) also having hepatic steatosis, increasing to 85% in individuals with grade III obesity (BMI = 40–59 kg/m^2^) [122]. ALT is a serum liver enzyme that is mainly concentrated in the cytosol of hepatocytes and is associated with metabolism, and indicates hepatocellular damage [123]. Being overweight has been established as a major risk factor for increased ALT levels and correlates with the high ALT levels observed in those with general obesity [124] and liver diseases [125]. Herein, we found that AST and ALT levels were significantly lower after the intervention period in the MIS treatment arm. Arora and colleagues (2022) demonstrated that ALT levels decrease in correlation with weight loss in patients with obesity and NAFLD, who followed a lifestyle intervention for six months [126]. Similarly, weight loss stemming from low energy intake has been shown to facilitate an improvement in AST [127]. Taken together, while we did not directly examine NAFLD and/or liver health via abdominal ultrasound, we observed a significant improvement in NAFLD-associated clinical biomarkers.

To examine the potential mechanisms of improved hepatic biomarkers, we performed additional molecular analysis. Growth differentiation factor-15 (GDF-15) is a novel cytokine member of the transforming growth factor β superfamily, whose expression increases in response to a variety of stimuli, including metabolic stress [128]. In the current study, 12 wks of MIS significantly increased circulating levels of GDF15 in the active group compared to those in the PLA treatment arm. Interestingly, higher levels of GDF15 have been associated with a higher prevalence of NAFLD in youth with obesity, as well as in overweight adults, with higher levels of circulating GDF15 linked to worsening of liver fibrosis [129,130]. However, this relationship is complex. Upon activation, the GDNF family receptor α–like (GFRAL), located in the hindbrain, can drive a desire to reduce food intake-making interpretation of circulating levels challenging [128]. To compound this challenge, evidence suggests that GFRAL and GDF15 are necessary for the weight loss benefits of metformin [131,132]. Recent studies in murine models have shown that GDF15 promotes weight loss by increasing energy expenditure via a GFRAL–β-adrenergic signaling and counteracts compensatory reductions in energy expenditure during periods of caloric restriction [133]. In humans, increases in GDF15 are seen with metformin-induced weight loss [132] and exercise training [134], suggesting that higher circulating GDF15 levels linked to metabolic disease are not causative, but rather because of the metabolic stress from obesity and NAFLD. In addition, an increase in GDF15 following weight loss in obese adults [135] further confirms the findings that an increase in GDF15 is associated with metabolic improvements following weight loss.

Another potential mechanism via which the MIS may elicit metabolic and hepatic benefits is via circulating miRNAs contained within extracellular vesicles. EVs are emerging mechanisms of intercellular communication through the release or shedding of vesicles by various cells [136,137,138], and can be critical for maintaining the health of adipocyte and hepatic cell homeostasis. Obesity [31] and NAFLD can alter miRNAs in adipose tissue and circulation, which may further exacerbate the disease pathology [139]. We found a significant reduction in EV-associated miR-34a and miR-122 following 12 wk intervention in the MIS treatment arm. In mice with fat-induced liver injury, both miR-34a and miR-122 have been correlated with hepatic histopathology [140]. In clinical studies, miR34a has been associated with NAFLD, with higher circulating miR34a levels prevalent in higher-severity disease phenotypes [141]. Similarly, miR-122 has also been associated with NAFLD, obesity, and insulin resistance [142], and the EVs of mice with fatty liver disease have a significant enrichment of mir-122 [143]. Interestingly, we observed an increased expression of miRNA-143 in the PLA treatment arm over the duration of the intervention period. miRNA-143 has been previously associated with fatty liver disease in obese adults undergoing bariatric surgery [144], as well as patients with NAFLD and coronary artery disease [145]. Taken together, a reduction in specific EV-associated miRNAs in the MIS treatment arm, in conjunction with observed improvements in other indices of hepatic function, suggests that altered intercellular communication following treatment may confer some hepatic and metabolic benefits.

Our examination of clinical hematology revealed a significant difference between the MIS and PLA treatment arms, with a significantly lower eosinophil count following 12 wks of MIS. Interestingly, eosinophil count has been previously shown to be positively correlated with BMI up to a plateau of 40 kg/m^2^, suggesting that obese patients have an elevated eosinophil count [146], with a similar relationship between patients with metabolic syndrome and elevated eosinophil count [147]. Future studies should examine whether the provision of MIS can alter allergic diseases such as asthma in obese patients.

An impairment of kidney function, specifically obesity-related glomerulopathy [148,149,150], suggests that obesity is associated with kidney disease [151,152]. Indeed, large-scale studies suggest that a BMI >30 kg/m^2^ with the concurrent risk factors of metabolic syndrome and insulin resistance is associated with a higher risk of developing kidney disease [153]. Consequently, the small but significant increase in creatinine in the MIS group, despite weight and fat loss, would seem paradoxical. First, it is important to note that the change in serum creatinine is not clinically significant, with the MIS treatment arm having a post-intervention serum creatine concentration of ~83 µmol/L. Further, central obesity has been associated with renal hyperfiltration [154] or an abnormally high glomerular filtration rate due to systemic hypertension, which often coexists in obese individuals [149,155]. Significant weight loss can improve glomerular flow hyperfiltration in obese individuals without overt renal disease [156]. Furthermore, it is well known that creatinine is also proportional to FFM, independent of GFR [150,157], and with the preservation of FFM concomitant with a lower total body mass, the ratio of fat-free mass to total mass may increase creatinine.

We acknowledge several limitations of the current study. A possible limitation of this study is that we did not have full control of physical activity and/or dietary intake during the experimental period. At the onset of the study, we provided simple diet information (the Canadian Food Guide) and only provided diet and activity guidelines at the inception to determine the potential effect of an MIS in a free-living scenario without intensive behavioral intervention. The lack of strict dietary and/or exercise controls and the impact of COVID-19 in the third inception cohort were also factors that may have contributed to the increase in body weight in the control group and may represent a potential attenuation of weight loss in the MIS group. Finally, the current study was conducted for 12 wks and thus we could not observe the sustainability of the effects over a longer period. Future studies should address interventions with longer durations.

## 5. Conclusions

In conclusion, the provision of 12 wks of MIS comprising ingredients known to facilitate mitochondrial function, increase lipid metabolism, and enhance body re-composition [52] was able to significantly reduce total body weight and fat mass, while preserving fat-free mass. Alterations in body composition were independent of alterations in energy intake or physical activity patterns. Further, we found that the MIS treatment arm had improved biochemical and EV-associated miRNA profiles associated with improved hepatic function. Future studies should examine the impact of physical activity in conjunction with MIS to achieve synergistic metabolic benefits.

## Figures and Tables

**Table 1:** Nutritional composition of the multi-ingredient supplement (MIS) and placebo (PLA) control supplement.

Ingredients	PLA	MIS
Forskolin (mg)	0	50
Green Coffee Bean Extract (mg)	0	500
Green Tea Extract (mg)	0	500
Beet Root Extract (mg)	0	500
α-Lipoic Acid (mg)	0	400
Vitamin E (IU)	0	200
Coenzyme Q10 (mg)	0	200
Microcrystalline Cellulose (g)	2.15	0

The quantities listed represent the complete daily serving of the MIS or PLA. Participants consumed servings of the supplement during the 12 wk intervention period (7 d/wk^−1^). MIS; multi-ingredient supplement, PLA; placebo control. PLA and MIS were both encapsulated in an opaque casing matched for appearance.

**Table 2:** Baseline participant characteristics in both treatment arms.

Variable (Unit)	Placebo (PLA; *n* = 27)	Active (MIS; *n* = 28)
Sex, *n* (M/F)	12/15	11/17
Age (years)	25.5 ± 1.5	26.4 ± 1.6
Body weight (kg)	87.8 ± 3.5	89.4 ± 2.8
BMI (kg/m^2^)	29.8 ± 1.0	31.1 ± 0.8
Waist-to-Height Ratio (a.u)	0.57 ± 0.01	0.59 ± 0.01
Body Fat percentage (%)	40.2 ± 1.7	40.3 ± 1.5
Fat mass (kg)	34.2 ± 2.3	34.6 ± 1.7
Fat-free mass (kg)	50.2 ± 0.1	51.4 ± 0.1

Data are presented as means ± SE. BMI: body mass index.

**Table 3:** Anthropometric measurements following 12 wks of treatment, adjusted for baseline variables.

Variable (Unit)	Placebo (*n*)	Active (*n*)	*p* Value
Weight (kg)	89.5 ± 0.5 (27)	86.4 ± 0.5 (28) ***	**<0.001**
BMI (kg/m^2^)	30.7 ± 0.2 (27)	29.7 ± 0.2 (28) ***	**<0.001**
Waist to Height ratio (a.u.)	0.60 ± 0.01 (27) *	0.58 ± 0.01 (28)	0.098
Total Fat-Free Mass (kg)	51.0 ± 0.4 (27)	50.7 ± 0.4 (28)	0.591
Total Fat Mass (kg) ^§,†^	35.4 ± 0.5 (27)	33.0 ± 0.5 (28) **	**<0.001**
BCI (a.u.)	1.57 ± 0.04 (27)	1.69 ± 0.04 (28) *	**0.034**
Arm Fat Mass (kg)	3.10 ± 0.06 (27)	3.01 ± 0.06 (28) *	0.237
Leg Fat Mass (kg)	13.5 ± 0.2 (27)	13.1 ± 0.2 (28) *	0.088
Trunk Fat Mass (kg)	17.4 ± 0.3 (27)	16.4 ± 0.3 (28) *	**0.039**
Android Fat (kg)	3.31 ± 0.06 (27)	3.14 ± 0.06 (28) *	0.051
Gynoid Fat (kg)	6.60 ± 0.09 (27)	6.22 ± 0.08 (28) **	**0.002**

Post-intervention data are presented as adjusted means ± SEs. Post-intervention variables were compared using one-way ANCOVA, with corresponding baseline variables as covariates. Within-group comparisons were made using a paired *t*-test. *; *p* < 0.05, **; *p* ≤ 0.01, ***; *p* ≤ 0.001, within-group comparison. ^§^ Between-group comparisons were made using the Mann–Whitney U test. ^†^ Within-group comparisons were made using the non-parametric sign rank test. BCI: body composition index; BMI: body mass index.

**Table 4:** Post-intervention clinical biochemistry, fasting glucose, and blood lipids following 12 wks of treatment, adjusted for baseline variables.

Variable (Unit)	Placebo (*n*)	Active (*n*)	*p* Value
*Clinical biochemistry*			
ALT (U/L)	30.3 ± 2.4 (21)	21.1 ± 2.4 (21) *	**0.008**
AST (U/L)	32.9 ± 1.6 (18)	27.3 ± 1.6 (18) *	**0.017**
GGT (U/L) ^‡^	29.3 ± 1.4 (21)	29.5 ± 1.4 (21)	0.913
CRP (mg/L) ^‡^	3.4 ± 1.0 (21)	4.2 ± 1.0 (21)	0.804
Creatinine (µmol/L) ^§,†^	80.0 ± 1.4 (21)	83.4 ± 1.4 (21) *	0.089
Bilirubin (µmol/L) ^‡^	12.3 ± 1.0 (21)	11.3 ± 1.0 (21)	0.362
*Fasting blood lipids & glucose homeostasis*			
Total Cholesterol (mmol/L)	4.8 ± 0.1 (21)	4.9 ± 0.1 (21)	0.526
LDL (mmol/L)	2.9 ± 0.1 (21)	3.0 ± 0.1 (21)	0.342
HDL (mmol/L)	1.3 ± 0.03 (21)	1.2 ± 0.03 (21)	0.311
Triglycerides (mmol/L)	1.4 ± 0.1 (21)	1.4 ± 0.1 (21)	0.924
Glucose (mmol/L)	5.0 ± 0.1 (21)	5.0 ± 0.1 (21)	0.942
Insulin (pmol/L)	80.1 ± 5.5 (21)	81.0 ± 5.5 (21)	0.908
HOMA IR (a.u.)	2.6 ± 0.2 (21)	2.6 ± 0.2 (21)	0.991

Post-intervention data are presented as adjusted means ± SEs. Post-intervention variables were compared using one-way ANCOVA, with corresponding baseline variables as covariates. Within-group comparisons were made using a paired *t*-test. ^‡^ Logarithmic transformation was required to achieve normality. ^§^ Between-group comparisons were made using the Mann–Whitney U test. ^†^ Within-group comparisons were made using the non-parametric sign rank test. * Denotes statistically significant (*p* < 0.05) within-group difference. HOMA-IR; Homeostatic Model Assessment for Insulin Resistance.

**Table 5:** Post-intervention hematology following 12 wks of treatment, adjusted for baseline variables.

Variable (Unit)	Placebo (*n*)	Active (*n*)	*p* Value
Hemoglobin	142.9 ± 1.19 (21)	143.7 ± 1.19 (21)	0.650
Hb (g/L)			
Hematocrit	0.4 ± 0.0 (21)	0.4 ± 0.0 (21)	0.977
HCT (%)			
Mean Corpuscular Volume	87.3 ± 0.3 (21)	86.9 ± 0.3 (21)	0.309
MCV (fL)			
Mean Corpuscular Hb	28.8 ± 0.1 (21)	28.9 ± 0.1 (21)	0.694
MCH (pg)			
Mean Corpuscular Hb Conc.	330.9 ± 1.5 (21)	331.3 ± 1.5 (21)	0.835
MCHC (g/L)			
Mean Platelet Volume ^§,†^	10.5 ± 0.1 (21)	10.6 ± 0.1 (21)	0.472
MPV (fL)			
Red Cell Distribution Width	12.9 ± 0.09 (21)	13.1 ± 0.09 (21)	0.172
RDW (%)			
Leukocyte Count	6.4 ± 0.3 (21)	6.5 ± 0.3 (21)	0.783
LKCS (×10^9^/L)			
Erythrocyte Count	5.0 ± 0.04 (21)	5.0 ± 0.04 (21)	0.864
ERCS (×10^12^/L)			
Platelet Count ^‡^	253.6 ± 6.5 (21)	254.9 ± 6.5 (21)	0.880
(×10^9^/L)			
Neutrophil Count	3.5 ± 0.2 (21)	3.8 ± 0.2 (21)	0.370
(×10^9^/L)			
Lymphocyte Count	2.1 ± 0.1 (21)	2.0 ± 0.1 (21)	0.273
(×10^9^/L)			
Monocyte Count ^‡^	0.5 ± 0.0 (21)	0.5 ± 0.0 (21)	0.555
(×10^9^/L)			
Eosinophil Count ^§,†^	0.19 ± 0.02 (21) *	0.13 ± 0.02 (21)	**0.007**
(×10^9^/L)			
Basophil Count ^§,†^	0.03 ± 0.01 (21)	0.04 ± 0.01 (21)	0.521
(×10^9^/L)			

Post-intervention data are presented as adjusted means ± SEs. Post-intervention variables were compared using one-way ANCOVA, with corresponding baseline variables as covariates. Within-group comparisons were made using a paired *t*-test. ^‡^ Logarithmic transformation was required to achieve normality. ^§^ Between-group comparisons were made using the Mann–Whitney U test. ^†^ Within-group comparisons were made using the non-parametric sign rank test. * Denotes statistically significant (*p* < 0.05) within-group difference.

**Table 6:** Post-intervention indices of physical health, function, and metabolism following 12 wks of treatment, adjusted for baseline variables.

Variable (Unit)	Placebo (*n*)	Active (*n*)	*p* Value
*Indices of health*			
Systolic Blood Pressure (mmHg)	121 ± 2 (21)	127 ± 2 (21)	0.08
Diastolic Blood Pressure (mmHg)	78 ± 2 (17)	81 ± 2 (17)	0.101
Resting Heart Rate (bpm)	77 ± 3 (21)	78 ± 3 (21)	0.655
Bone mineral density (g/cm^2^)	1.24 ± 0.01 (27)	1.24 ± 0.01 (28)	0.734
*Indices of physical fitness*			
Relative VO_2peak_ ^§,†^ (mL/kg/min^−1^)	29.6 ± 1.2 (19)	30.1 ± 1.1 (21)	0.526
Relative Grip Strength (kg/kg bw^−1^)	0.42 ± 0.01 (21) *	0.47 ± 0.01 (21) *	**0.002**
*Indices of metabolic rate*			
Resting metabolic rate (Kcal/day)	1808.3 ± 44.5 (21)	1827.6 ± 44.5 (21)	0.761
Estimated metabolic rate (Kcal/day)	1736.3 ± 5.0 (27)	1705.0 ± 4.9 (28) *	**<0.001**

Post-intervention data are presented as adjusted means ± SEs. Post-intervention variables were compared using one-way ANCOVA, with corresponding baseline variables as covariates. Within-group comparisons were made using a paired *t*-test. ^§^ Between-group comparisons were made using the Mann–Whitney U test. ^†^ Within-group comparisons were made using the non-parametric sign rank test. * Denotes statistically significant (*p* < 0.05) within-group difference.

**Table 7:** Post-intervention indices of molecular markers of inflammation, metabolism, and antioxidant capacity following 12 wks of treatment, adjusted for baseline variables.

Variable (Unit)	Placebo (*n*)	Active (*n*)	*p* Value
GDF15 (pg/mL)	411.1 ± 17.0 (20)	466.0 ± 17.0 (20) *	**0.028**
FGF21 (µg/mL) ^§,†^	253.5 ± 48.0 (20)	313.4 ± 48.0 (20)	0.192
IL-6 (pg/mL) ^§,†^	110.1 ± 11.3 (18)	103.8 ± 10.7 (20)	0.633
Leptin (ng/mL)	28.5 ± 2.0 (20)	25.1 ± 1.9 (19)	0.225
ORAC (µM trolox equivalents/mL)	129.0 ± 2.9 (20)	136.6 ± 2.9 (20) *	0.072
CoQ10 (µg/mL) ^§,†^	1405.3 ± 58.9 (16)	1544.5 ± 51.2 (21) *	0.137

Post-intervention data are presented as adjusted means ± SEs. Post-intervention variables were compared using one-way ANCOVA, with corresponding baseline variables as covariates. Within-group comparisons were made using a paired *t*-test. ^§^ Between-group comparisons were made using the Mann–Whitney U test. ^†^ Within-group comparisons were made using the non-parametric sign rank test. GDF15, growth differentiation factor 15; FGF21, fibroblast growth factor 21; IL-6, interleukin 6; ORAC, Oxygen Radical Absorbance Capacity. * Denotes statistically significant (*p* < 0.05) within-group difference relative to Day 0 (baseline).

## Data Availability

Not applicable.

## Supplementary Materials

The following supporting information can be downloaded at: https://www.mdpi.com/article/10.3390/nu15173693/s1, Figure S1: Enrollment process. Figure S2: Study Schematic. Table S1: Drug Extract Ratio (DER) and solvents. Table S2: miRNA assay IDs.
