# Supplementary material for: Novel Multi-Ingredient Supplement Facilitates Weight Loss and Improves Body Composition in Overweight and Obese Individuals: A Randomized, Double-Blind, Placebo-Controlled Clinical Trial
Source: Nutrients. 2023 Aug 23;15(17):3693. doi: 10.3390/nu15173693 (PMC10490028; doi:10.3390/nu15173693)
Supplement: Supplementary file 1 [file nutrients-15-03693-s001.zip › Supplementary Materials OBIII (8.20.23).pdf]

*SUPPLEMENTARY MATERIALS*

# *Article*: Novel Multi-Ingredient Supplement Facilitates Weight Loss and Improves Body Composition in Overweight and Obese Individuals: A Randomized, Double-Blind, Placebo-Controlled Clinical Trial

**Joshua P. Nederveen [1], Alexander J. Mastrolonardo [1], Donald Xhuti [1], Alessia Di Carlo [1], Katherine Manta [1], Matthew R. Fuda [1] and Mark A. Tarnopolsky [1,2,*]**

[1] Department of Pediatrics, Faculty of Health Sciences, McMaster University Medical Center (MUMC), Hamilton, ON L8N 3Z5, Canada
[2] Exerkine Corporation, McMaster University Medical Center (MUMC), Hamilton, ON L8N 3Z5, Canada
* Correspondence: tarnopol@mcmaster.ca

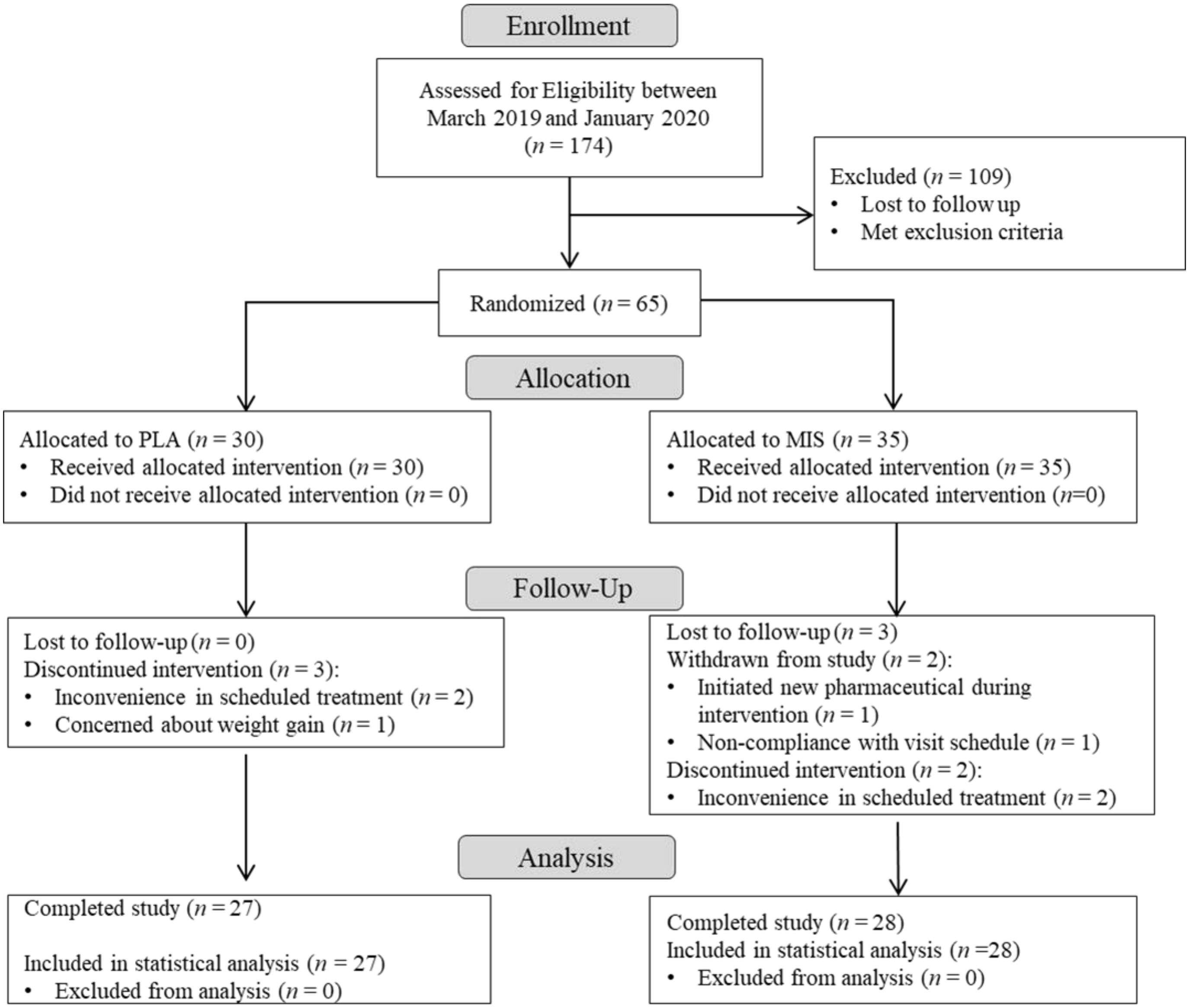


**Figure S1.** Flowchart of the enrollment process and study progression.

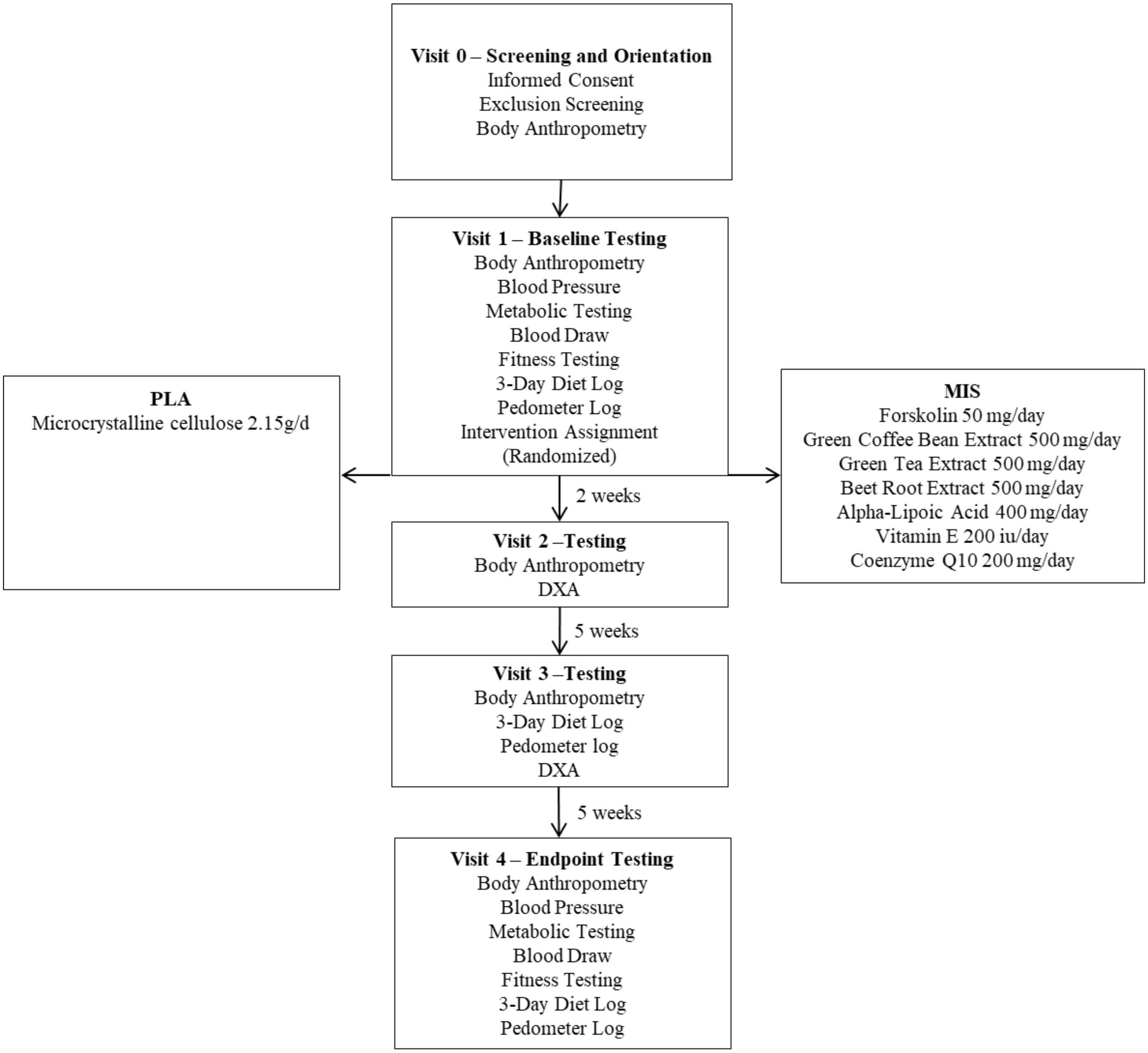


**Figure S2.** Study Schematic.

**Table S1.** Scientific names of the plant-based MIS components

| MIS Component | Scientific Name | Plant Part | DER | Extraction Solvent |
|---|---|---|---|---|
| Green Coffee Bean Extract§ | *Coffea Arabica* | Seed | 12:1 | Water, ethanol |
| Green Tea Extract‡ | *Camellia Sinensis* | Leaf | 30:1 | Water |
| Forskohlii Extract† | *Coleus Forskohlii* or *Plectranthus Barbatus* | Root | 100:1 | Toulene* |
| Beet Root Extract | *Beta Vulgaris* | Root | 15:1 | Water |

DER: drug extract ratio. MIS: multi-ingredient supplement. *; manufacturer process utilized hexane and ethanol, §; standardized to 45% chlorogenic acids, ‡; standardized to 50% epigallocatechin-3-gallate (EGCG), †; standardized to 40% forskolin.

**Table S2.** miRNA Assay IDs

| miRNA Species | Assay ID |
|---|---|
| hsa-miR-29a-3p | 478587_mir |
| hsa-miR-34a-5p | 478048_mir |
| hsa-miR-122-5p | 477855_mir |
| hsa-miR-143-3p | 477912_mir |
| hsa-miR-146a-5p | 478399_mir |
| cel-miR-54-3p | 478410_mir |

miRNA: microRNA
